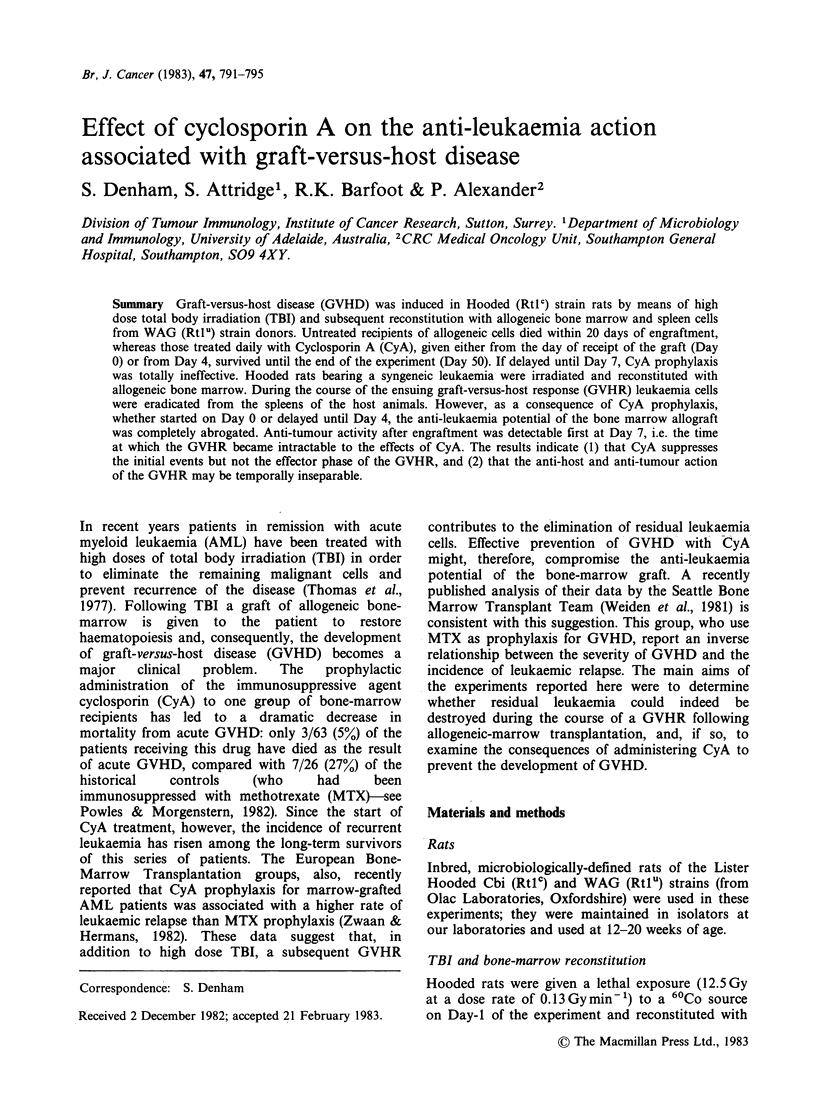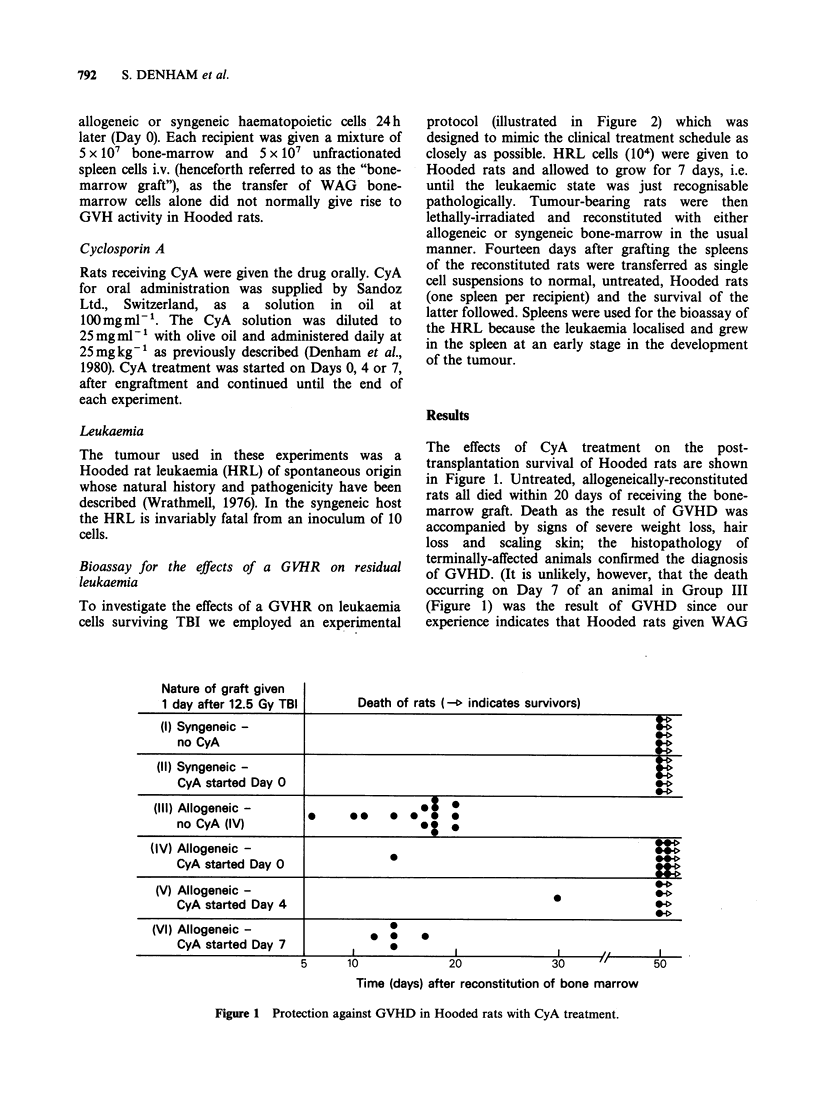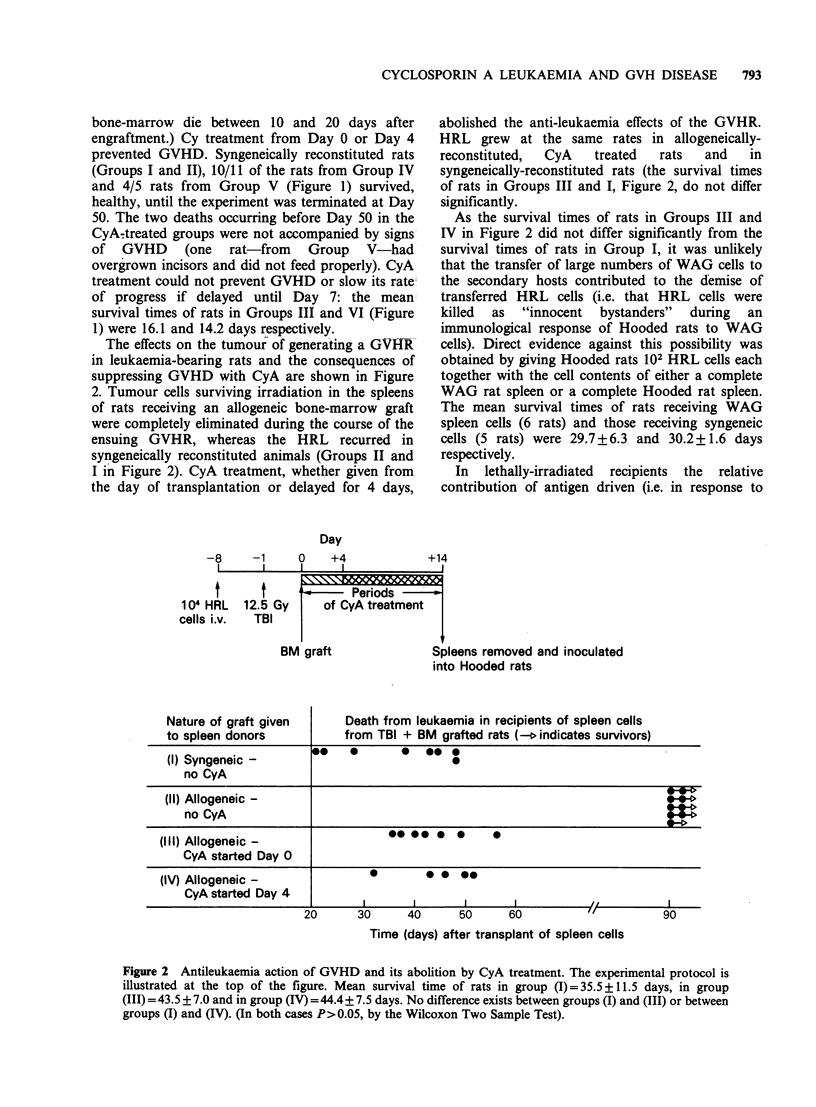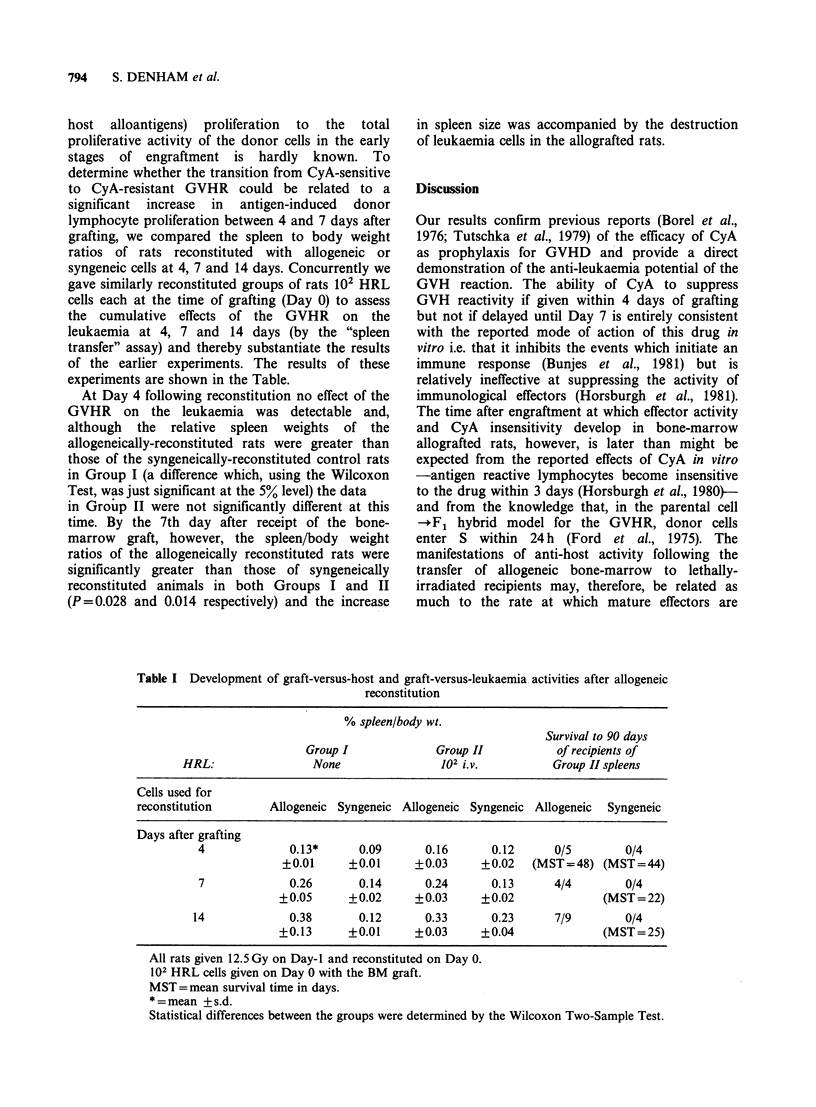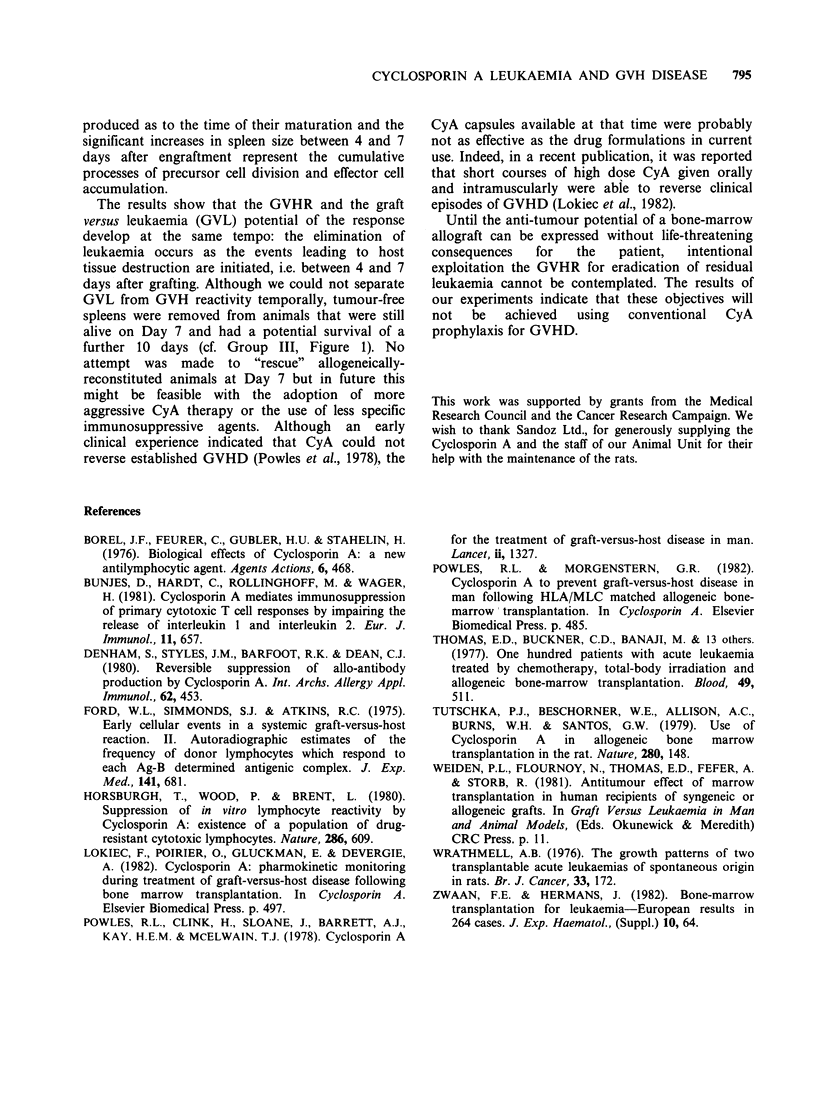# Effect of cyclosporin A on the anti-leukaemia action associated with graft-versus-host disease.

**DOI:** 10.1038/bjc.1983.132

**Published:** 1983-06

**Authors:** S. Denham, S. Attridge, R. K. Barfoot, P. Alexander

## Abstract

Graft-versus-host disease (GVHD) was induced in Hooded (Rt1c) strain rats by means of high dose total body irradiation (TBI) and subsequent reconstitution with allogeneic bone marrow and spleen cells from WAG (Rt1u) strain donors. Untreated recipients of allogeneic cells died within 20 days of engraftment, whereas those treated daily with Cyclosporin A (CyA), given either from the day receipt of the graft (Day 0) or from Day 4, survived until the end of the experiment (Day 50). If delayed until Day 7, CyA prophylaxis was totally ineffective. Hooded rats bearing a syngeneic leukaemia were irradiated and reconstituted with allogeneic bone marrow. During the course of the ensuing graft-versus-host response (GVHR) leukaemia cells were eradicated from the spleens of the host animals. However, as a consequence of CyA prophylaxis, whether started on Day 0 or delayed until Day 4, the anti-leukaemia potential of the bone marrow allograft was completely abrogated. Anti-tumour activity after engraftment was detectable first at Day 7, i.e. the time at which the GVHR became intractable to the effects of CyA. The results indicate (1) that CyA suppresses the initial events but not the effector phase of the GVHR, and (2) that the anti-host and anti-tumour action of the GVHR may be temporally inseparable.


*Br, J. Cancer* (1983), **47**, 791–795

### Effect of cyclosporin A on the anti-leukaemia action associated with graft-versus-host disease

S. Denham, S. Attridge[1], R.K. Barfoot & P. Alexander[2]

*Division of Tumour Immunology, Institute of Cancer Research, Sutton, Surrey. [1]Department of Microbiology and Immunology, University of Adelaide, Australia, [2]CRC Medical Oncology Unit, Southampton General Hospital, Southampton, SO9 4XY.*

**Summary** Graft-versus-host disease (GVHD) was induced in Hooded ($Rtl^c$) strain rats by means of high dose total body irradiation (TBI) and subsequent reconstitution with allogeneic bone marrow and spleen cells from WAG ($Rtl^u$) strain donors. Untreated recipients of allogeneic cells died within 20 days of engraftment, whereas those treated daily with Cyclosporin A (CyA), given either from the day of receipt of the graft (Day 0) or from Day 4, survived until the end of the experiment (Day 50). If delayed until Day 7, CyA prophylaxis was totally ineffective. Hooded rats bearing a syngeneic leukaemia were irradiated and reconstituted with allogeneic bone marrow. During the course of the ensuing graft-versus-host response (GVHR) leukaemia cells were eradicated from the spleens of the host animals. However, as a consequence of CyA prophylaxis, whether started on Day 0 or delayed until Day 4, the anti-leukaemia potential of the bone marrow allograft was completely abrogated. Anti-tumour activity after engraftment was detectable first at Day 7, i.e. the time at which the GVHR became intractable to the effects of CyA. The results indicate (1) that CyA suppresses the initial events but not the effector phase of the GVHR, and (2) that the anti-host and anti-tumour action of the GVHR may be temporally inseparable.

In recent years patients in remission with acute myeloid leukaemia (AML) have been treated with high doses of total body irradiation (TBI) in order to eliminate the remaining malignant cells and prevent recurrence of the disease (Thomas *et al.*, 1977). Following TBI a graft of allogeneic bone-marrow is given to the patient to restore haematopoiesis and, consequently, the development of graft-*versus*-host disease (GVHD) becomes a major clinical problem. The prophylactic administration of the immunosuppressive agent cyclosporin (CyA) to one group of bone-marrow recipients has led to a dramatic decrease in mortality from acute GVHD: only 3/63 (5%) of the patients receiving this drug have died as the result of acute GVHD, compared with 7/26 (27%) of the historical controls (who had been immunosuppressed with methotrexate (MTX)—see Powles & Morgenstern, 1982). Since the start of CyA treatment, however, the incidence of recurrent leukaemia has risen among the long-term survivors of this series of patients. The European Bone-Marrow Transplantation groups, also, recently reported that CyA prophylaxis for marrow-grafted AML patients was associated with a higher rate of leukaemic relapse than MTX prophylaxis (Zwaan & Hermans, 1982). These data suggest that, in addition to high dose TBI, a subsequent GVHR contributes to the elimination of residual leukaemia cells. Effective prevention of GVHD with CyA might, therefore, compromise the anti-leukaemia potential of the bone-marrow graft. A recently published analysis of their data by the Seattle Bone Marrow Transplant Team (Weiden *et al.*, 1981) is consistent with this suggestion. This group, who use MTX as prophylaxis for GVHD, report an inverse relationship between the severity of GVHD and the incidence of leukaemic relapse. The main aims of the experiments reported here were to determine whether residual leukaemia could indeed be destroyed during the course of a GVHR following allogeneic-marrow transplantation, and, if so, to examine the consequences of administering CyA to prevent the development of GVHD.

Correspondence: S. Denham

Received 2 December 1982; accepted 21 February 1983.

### Materials and methods

### *Rats*

Inbred, microbiologically-defined rats of the Lister Hooded Cbi ($Rtl^c$) and WAG ($Rtl^u$) strains (from Olac Laboratories, Oxfordshire) were used in these experiments; they were maintained in isolators at our laboratories and used at 12–20 weeks of age.

### *TBI and bone-marrow reconstitution*

Hooded rats were given a lethal exposure (12.5 Gy at a dose rate of $0.13\,Gy\,min^{-1}$) to a $^{60}Co$ source on Day-1 of the experiment and reconstituted with

© The Macmillan Press Ltd., 1983

allogeneic or syngeneic haematopoietic cells 24 h later (Day 0). Each recipient was given a mixture of $5 \times 10^7$ bone-marrow and $5 \times 10^7$ unfractionated spleen cells i.v. (henceforth referred to as the "bone-marrow graft"), as the transfer of WAG bone-marrow cells alone did not normally give rise to GVH activity in Hooded rats.

*Cyclosporin A*

Rats receiving CyA were given the drug orally. CyA for oral administration was supplied by Sandoz Ltd., Switzerland, as a solution in oil at $100 \, mg \, ml^{-1}$. The CyA solution was diluted to $25 \, mg \, ml^{-1}$ with olive oil and administered daily at $25 \, mg \, kg^{-1}$ as previously described (Denham *et al.*, 1980). CyA treatment was started on Days 0, 4 or 7, after engraftment and continued until the end of each experiment.

*Leukaemia*

The tumour used in these experiments was a Hooded rat leukaemia (HRL) of spontaneous origin whose natural history and pathogenicity have been described (Wrathmell, 1976). In the syngeneic host the HRL is invariably fatal from an inoculum of 10 cells.

*Bioassay for the effects of a GVHR on residual leukaemia*

To investigate the effects of a GVHR on leukaemia cells surviving TBI we employed an experimental protocol (illustrated in Figure 2) which was designed to mimic the clinical treatment schedule as closely as possible. HRL cells ($10^4$) were given to Hooded rats and allowed to grow for 7 days, i.e. until the leukaemic state was just recognisable pathologically. Tumour-bearing rats were then lethally-irradiated and reconstituted with either allogeneic or syngeneic bone-marrow in the usual manner. Fourteen days after grafting the spleens of the reconstituted rats were transferred as single cell suspensions to normal, untreated, Hooded rats (one spleen per recipient) and the survival of the latter followed. Spleens were used for the bioassay of the HRL because the leukaemia localised and grew in the spleen at an early stage in the development of the tumour.

**Results**

The effects of CyA treatment on the post-transplantation survival of Hooded rats are shown in Figure 1. Untreated, allogeneically-reconstituted rats all died within 20 days of receiving the bone-marrow graft. Death as the result of GVHD was accompanied by signs of severe weight loss, hair loss and scaling skin; the histopathology of terminally-affected animals confirmed the diagnosis of GVHD. (It is unlikely, however, that the death occurring on Day 7 of an animal in Group III (Figure 1) was the result of GVHD since our experience indicates that Hooded rats given WAG

**Figure 1** Protection against GVHD in Hooded rats with CyA treatment.

bone-marrow die between 10 and 20 days after engraftment.) Cy treatment from Day 0 or Day 4 prevented GVHD. Syngeneically reconstituted rats (Groups I and II), 10/11 of the rats from Group IV and 4/5 rats from Group V (Figure 1) survived, healthy, until the experiment was terminated at Day 50. The two deaths occurring before Day 50 in the CyA-treated groups were not accompanied by signs of GVHD (one rat—from Group V—had overgrown incisors and did not feed properly). CyA treatment could not prevent GVHD or slow its rate of progress if delayed until Day 7: the mean survival times of rats in Groups III and VI (Figure 1) were 16.1 and 14.2 days respectively.

The effects on the tumour of generating a GVHR in leukaemia-bearing rats and the consequences of suppressing GVHD with CyA are shown in Figure 2. Tumour cells surviving irradiation in the spleens of rats receiving an allogeneic bone-marrow graft were completely eliminated during the course of the ensuing GVHR, whereas the HRL recurred in syngeneically reconstituted animals (Groups II and I in Figure 2). CyA treatment, whether given from the day of transplantation or delayed for 4 days, abolished the anti-leukaemia effects of the GVHR. HRL grew at the same rates in allogeneically-reconstituted, CyA treated rats and in syngeneically-reconstituted rats (the survival times of rats in Groups III and I, Figure 2, do not differ significantly.

As the survival times of rats in Groups III and IV in Figure 2 did not differ significantly from the survival times of rats in Group I, it was unlikely that the transfer of large numbers of WAG cells to the secondary hosts contributed to the demise of transferred HRL cells (i.e. that HRL cells were killed as "innocent bystanders" during an immunological response of Hooded rats to WAG cells). Direct evidence against this possibility was obtained by giving Hooded rats $10^2$ HRL cells each together with the cell contents of either a complete WAG rat spleen or a complete Hooded rat spleen. The mean survival times of rats receiving WAG spleen cells (6 rats) and those receiving syngeneic cells (5 rats) were $29.7 \pm 6.3$ and $30.2 \pm 1.6$ days respectively.

In lethally-irradiated recipients the relative contribution of antigen driven (i.e. in response to

**Figure 2** Antileukaemia action of GVHD and its abolition by CyA treatment. The experimental protocol is illustrated at the top of the figure. Mean survival time of rats in group (I) $= 35.5 \pm 11.5$ days, in group (III) $= 43.5 \pm 7.0$ and in group (IV) $= 44.4 \pm 7.5$ days. No difference exists between groups (I) and (III) or between groups (I) and (IV). (In both cases $P > 0.05$, by the Wilcoxon Two Sample Test).

host alloantigens) proliferation to the total proliferative activity of the donor cells in the early stages of engraftment is hardly known. To determine whether the transition from CyA-sensitive to CyA-resistant GVHR could be related to a significant increase in antigen-induced donor lymphocyte proliferation between 4 and 7 days after grafting, we compared the spleen to body weight ratios of rats reconstituted with allogeneic or syngeneic cells at 4, 7 and 14 days. Concurrently we gave similarly reconstituted groups of rats $10^2$ HRL cells each at the time of grafting (Day 0) to assess the cumulative effects of the GVHR on the leukaemia at 4, 7 and 14 days (by the "spleen transfer" assay) and thereby substantiate the results of the earlier experiments. The results of these experiments are shown in the Table.

At Day 4 following reconstitution no effect of the GVHR on the leukaemia was detectable and, although the relative spleen weights of the allogeneically-reconstituted rats were greater than those of the syngeneically-reconstituted control rats in Group I (a difference which, using the Wilcoxon Test, was just significant at the 5% level) the data in Group II were not significantly different at this time. By the 7th day after receipt of the bone-marrow graft, however, the spleen/body weight ratios of the allogeneically reconstituted rats were significantly greater than those of syngeneically reconstituted animals in both Groups I and II ($P=0.028$ and 0.014 respectively) and the increase in spleen size was accompanied by the destruction of leukaemia cells in the allografted rats.

### Discussion

Our results confirm previous reports (Borel *et al.*, 1976; Tutschka *et al.*, 1979) of the efficacy of CyA as prophylaxis for GVHD and provide a direct demonstration of the anti-leukaemia potential of the GVH reaction. The ability of CyA to suppress GVH reactivity if given within 4 days of grafting but not if delayed until Day 7 is entirely consistent with the reported mode of action of this drug *in vitro* i.e. that it inhibits the events which initiate an immune response (Bunjes *et al.*, 1981) but is relatively ineffective at suppressing the activity of immunological effectors (Horsburgh *et al.*, 1981). The time after engraftment at which effector activity and CyA insensitivity develop in bone-marrow allografted rats, however, is later than might be expected from the reported effects of CyA *in vitro* —antigen reactive lymphocytes become insensitive to the drug within 3 days (Horsburgh *et al.*, 1980)— and from the knowledge that, in the parental cell $\rightarrow F_1$ hybrid model for the GVHR, donor cells enter S within 24 h (Ford *et al.*, 1975). The manifestations of anti-host activity following the transfer of allogeneic bone-marrow to lethally-irradiated recipients may, therefore, be related as much to the rate at which mature effectors are

**Table I** Development of graft-versus-host and graft-versus-leukaemia activities after allogeneic reconstitution

| | *% spleen/body wt.* | | | | *Survival to 90 days of recipients of Group II spleens* | |
|---|---|---|---|---|---|---|
| *HRL:* | *Group I None* | | *Group II $10^2$ i.v.* | | | |
| Cells used for reconstitution | Allogeneic | Syngeneic | Allogeneic | Syngeneic | Allogeneic | Syngeneic |
| Days after grafting | | | | | | |
| 4 | 0.13* ±0.01 | 0.09 ±0.01 | 0.16 ±0.03 | 0.12 ±0.02 | 0/5 (MST=48) | 0/4 (MST=44) |
| 7 | 0.26 ±0.05 | 0.14 ±0.02 | 0.24 ±0.03 | 0.13 ±0.02 | 4/4 | 0/4 (MST=22) |
| 14 | 0.38 ±0.13 | 0.12 ±0.01 | 0.33 ±0.03 | 0.23 ±0.04 | 7/9 | 0/4 (MST=25) |

All rats given 12.5 Gy on Day-1 and reconstituted on Day 0.
$10^2$ HRL cells given on Day 0 with the BM graft.
MST = mean survival time in days.
* = mean ±s.d.
Statistical differences between the groups were determined by the Wilcoxon Two-Sample Test.

produced as to the time of their maturation and the significant increases in spleen size between 4 and 7 days after engraftment represent the cumulative processes of precursor cell division and effector cell accumulation.

The results show that the GVHR and the graft *versus* leukaemia (GVL) potential of the response develop at the same tempo: the elimination of leukaemia occurs as the events leading to host tissue destruction are initiated, i.e. between 4 and 7 days after grafting. Although we could not separate GVL from GVH reactivity temporally, tumour-free spleens were removed from animals that were still alive on Day 7 and had a potential survival of a further 10 days (cf. Group III, Figure 1). No attempt was made to "rescue" allogeneically-reconstituted animals at Day 7 but in future this might be feasible with the adoption of more aggressive CyA therapy or the use of less specific immunosuppressive agents. Although an early clinical experience indicated that CyA could not reverse established GVHD (Powles *et al.*, 1978), the CyA capsules available at that time were probably not as effective as the drug formulations in current use. Indeed, in a recent publication, it was reported that short courses of high dose CyA given orally and intramuscularly were able to reverse clinical episodes of GVHD (Lokiec *et al.*, 1982).

Until the anti-tumour potential of a bone-marrow allograft can be expressed without life-threatening consequences for the patient, intentional exploitation the GVHR for eradication of residual leukaemia cannot be contemplated. The results of our experiments indicate that these objectives will not be achieved using conventional CyA prophylaxis for GVHD.

This work was supported by grants from the Medical Research Council and the Cancer Research Campaign. We wish to thank Sandoz Ltd., for generously supplying the Cyclosporin A and the staff of our Animal Unit for their help with the maintenance of the rats.

### References

BOREL, J.F., FEURER, C., GUBLER, H.U. & STAHELIN, H. (1976). Biological effects of Cyclosporin A: a new antilymphocytic agent. *Agents Actions*, **6**, 468.

BUNJES, D., HARDT, C., ROLLINGHOFF, M. & WAGER, H. (1981). Cyclosporin A mediates immunosuppression of primary cytotoxic T cell responses by impairing the release of interleukin 1 and interleukin 2. *Eur. J. Immunol.*, **11**, 657.

DENHAM, S., STYLES, J.M., BARFOOT, R.K. & DEAN, C.J. (1980). Reversible suppression of allo-antibody production by Cyclosporin A. *Int. Archs. Allergy Appl. Immunol.*, **62**, 453.

FORD, W.L., SIMMONDS, S.J. & ATKINS, R.C. (1975). Early cellular events in a systemic graft-versus-host reaction. II. Autoradiographic estimates of the frequency of donor lymphocytes which respond to each Ag-B determined antigenic complex. *J. Exp. Med.*, **141**, 681.

HORSBURGH, T., WOOD, P. & BRENT, L. (1980). Suppression of *in vitro* lymphocyte reactivity by Cyclosporin A: existence of a population of drug-resistant cytotoxic lymphocytes. *Nature*, **286**, 609.

LOKIEC, F., POIRIER, O., GLUCKMAN, E. & DEVERGIE, A. (1982). Cyclosporin A: pharmokinetic monitoring during treatment of graft-versus-host disease following bone marrow transplantation. In *Cyclosporin A*. Elsevier Biomedical Press. p. 497.

POWLES, R.L., CLINK, H., SLOANE, J., BARRETT, A.J., KAY, H.E.M. & MCELWAIN, T.J. (1978). Cyclosporin A for the treatment of graft-versus-host disease in man. *Lancet*, **ii**, 1327.

POWLES, R.L. & MORGENSTERN, G.R. (1982). Cyclosporin A to prevent graft-versus-host disease in man following HLA/MLC matched allogeneic bone-marrow transplantation. In *Cyclosporin A*. Elsevier Biomedical Press. p. 485.

THOMAS, E.D., BUCKNER, C.D., BANAJI, M. & 13 others. (1977). One hundred patients with acute leukaemia treated by chemotherapy, total-body irradiation and allogeneic bone-marrow transplantation. *Blood*, **49**, 511.

TUTSCHKA, P.J., BESCHORNER, W.E., ALLISON, A.C., BURNS, W.H. & SANTOS, G.W. (1979). Use of Cyclosporin A in allogeneic bone marrow transplantation in the rat. *Nature*, **280**, 148.

WEIDEN, P.L., FLOURNOY, N., THOMAS, E.D., FEFER, A. & STORB, R. (1981). Antitumour effect of marrow transplantation in human recipients of syngeneic or allogeneic grafts. In *Graft Versus Leukaemia in Man and Animal Models*, (Eds. Okunewick & Meredith) CRC Press. p. 11.

WRATHMELL, A.B. (1976). The growth patterns of two transplantable acute leukaemias of spontaneous origin in rats. *Br. J. Cancer*, **33**, 172.

ZWAAN, F.E. & HERMANS, J. (1982). Bone-marrow transplantation for leukaemia—European results in 264 cases. *J. Exp. Haematol.*, (Suppl.) **10**, 64.